# Radiologic Diagnosis and Hospitalization among Children with Severe Community Acquired Pneumonia: A Prospective Cohort Study

**DOI:** 10.1155/2019/6202405

**Published:** 2019-01-09

**Authors:** Meiron Hassen, Alemayehu Toma, Mulugeta Tesfay, Eyoel Degafu, Solomon Bekele, Freshwork Ayalew, Abel Gedefaw, Birkneh Tilahun Tadesse

**Affiliations:** ^1^Adare General Hospital, Pediatrics Unit, Hawassa, Ethiopia; ^2^Hawassa University, College of Medicine and Health Sciences, Department of Pediatrics and Child health, Hawassa, P.O. Box 1560, Ethiopia; ^3^Hawassa University, College of Medicine and Health Sciences, School of Pharmacy, Department of Pharmacology and Toxicology, Hawassa, P.O. Box 1560, Ethiopia; ^4^Bete-Abrham Primary Hospital, Department of Radiology, Hawassa, P.O. Box 851, Ethiopia; ^5^Alatyon General Hospital, Department of Radiology, Hawassa, P.O. Box 1267, Ethiopia; ^6^Hawassa University, College of Medicine and Health Sciences, Department of Radiology, Hawassa, P.O. Box 1560, Ethiopia; ^7^Hawassa University, College of Medicine and Health Sciences, School of Laboratory Medicine, Hawassa, P.O. Box 1560, Ethiopia; ^8^Hawassa University, College of Medicine and Health Sciences, Department of Gynecology and Obstetrics, Hawassa, P.O. Box 1560, Ethiopia

## Abstract

**Objectives:**

This study was designed to assess the role of chest radiography for the diagnosis of pneumonia and assess the association of clinical characteristics with radiologic findings and predictors of hospitalization among children with severe community acquired pneumonia.

**Methods:**

A prospective study was conducted on 122 children between ages of 3 month and 14 years admitted to pediatric emergency unit with diagnosis of severe pneumonia from September 1^st^ to November 30^th^, 2017. Eligible children were subjected to chest radiography which was read by two senior radiologists independently (*R1* and* R2*). Disagreements between* R1* and* R2 *were resolved by a third senior radiologist (*R3*). Level of agreement between radiologists was assessed using Cohen's kappa coefficient. Clinical and laboratory parameters which could explain the variability in the duration of hospital stay were assessed using a linear regression mode. Independent predictors were assessed using multiple linear regression.

**Results:**

The median age of the cohort was 10.0 months (interquartile range (IQR): 6.75–24.0); 76 (62.3%) were male. Nearly half, 63 (51.6%) did not have radiologic evidence of pneumonia. There was low level of agreement between* R1* and* R2* in reporting consolidation (kappa=0.435, p-value≤0.001), haziness (kappa=0.375, p-value≤0.001), and infiltration (kappa=0.267, p-value=0.008). Children with higher recorded temperature were more likely to have radiologic abnormalities suggesting pneumonia (p-value=0.033). The median duration of hospitalization was 3 days (IQR: 1-4 days); 118 (96.7%) were discharged with improvement. Height-for-age z-score (Coef.=0.203, R^2^=0.041, p-value=0.027); and hemoglobin level (Coef.=-0.249, R^2^=0.062, p-value=0.006) explained 4.1% and 6.2% of the variability in the duration of hospital stay, respectively.

**Conclusion:**

Radiologic evidence of pneumonia was absent in half of the children with severe pneumonia. There was low agreement between senior radiologists in reporting chest radiographic findings, potentially necessitating harmonization activities to uniformly implement the WHO guidelines in reading chest radiographs.

## 1. Background

Worldwide, pneumonia was responsible for 15% of childhood deaths in 2016, with highest incidence in developing countries [1]. The global annual incidence of pneumonia is 150 to 156 million cases, accounting for approximately 10-20 million hospitalizations [1]. In Ethiopia, acute respiratory tract infections, particularly pneumonia, are the leading causes of child mortality and morbidity. Pneumonia accounts for 18% of childhood deaths in Ethiopia [2]. Typically, cough, pleuritic chest pain, fever, fatigue, and loss of appetite are presenting symptoms of pneumonia. Children and the elderly with pneumonia have varying presenting features, which include headache, nausea, abdominal pain, and absence of one or more of the prototypical symptoms [3, 4].

The diagnosis of pneumonia is based mainly on clinical parameters including respiratory symptoms and signs, which incur no cost and are sensitive, but are also nonspecific and could lead to unnecessary prescription of antibiotics and drug resistance. According to World Health Organization (WHO) guidelines, severe pneumonia is diagnosed when there is cough and fast breathing (defined based on respiratory rate for age) with one or two of the severity signs including chest in-drawing (in infants <2 months of age), grunting, cyanosis, inability to feed, lethargy, or convulsion [5]. Chest X-ray (CXR) has been the main stay modality in the investigation of chest infection since its invention in the late 19^th^ century, despite the advances in imaging modalities [6]. The WHO recommends CXR for all patients clinically diagnosed with severe pneumonia at tertiary centers [7–11]. Chest radiograph improves the diagnosis of pediatric community acquired pneumonia to a certain degree and may prevent overtreatment with antibiotics [12]. However, it is important to understand that interpretation is subject to perceptual and cognitive limitation and errors [13].

The interpretation of radiographs is difficult in young children and is affected by the radiographer's experience and the amount of clinical information available. Additionally, chest radiography cannot reliably distinguish between viral and bacterial pneumonia and is often unable to detect early changes of pneumonia [6, 11, 14]. Other drawbacks of chest radiography include exposure to ionizing radiation, cost, the time and space used, and the need to wait from the radiograph and to see the clinician again. Moreover, chest radiography has a low sensitivity compared to advanced imaging tools like lung ultrasound, computed tomography, and magnetic resonance imaging [14–16].

Timely diagnosis and management of severe pneumonia, and shorter hospital stay is crucial to decrease childhood morbidity and mortality in resource limited settings. Prolonged hospitalization could be associated with unfavorable outcomes and is also a cost burden to the public [17–21]. Several preventable risk factors have been reported to be associated with prolonged hospitalization among children with severe community acquired pneumonia. Failing to exclusively breastfeed for the first 6 months, inappropriate complimentary feeding, anemia, malnutrition, exposure to parental smoking, inadequate antibiotic use, lack of awareness of parents, overuse of nonsteroidal anti-inflammatory drugs, and indoor air pollution contribute to prolonging duration of hospitalization [22, 23]. History of full vaccination on the other hand decreased length of stay in the hospital [24].

Ethiopia is one of the high burden countries for childhood morbidity and mortality associated with severe pneumonia. It is common practice for clinicians working in hospitals to routinely request CXR for the diagnosis of pneumonia. Chest radiographic films are usually read by radiologists. However, there are no data to assess the validity of the readings by the radiologists and the role of CXR in treating children with pneumonia. In the current study three clinical questions were posed: (1) What is the level of agreement between two radiologists in reading CXR films of children with severe pneumonia? (2) What is the magnitude and predictors of radiologic pneumonia in children? (3) What are the predictors of duration of hospitalization among children with severe pneumonia in Ethiopia?

## 2. Methods and Materials

### 2.1. Study Setting, Design, and Research Questions

The study was conducted in Hawassa University Specialized Comprehensive Hospital (HUCSH), which is found in the southern region of Ethiopia. HUSCH is the first referral hospital established in the region serving as a teaching hospital for the College of Medicine and Health Science of Hawassa University, with a catchment population of 10-12 million. It receives about 43,384 patients annually. The hospital gives free service for patients with high priority pediatric conditions, including severe pneumonia.

A prospective short cohort study was conducted from September 1^st^ to November 30^th^, 2017. Children with a clinical diagnosis of severe community acquired pneumonia were followed from admission until discharge. All pediatric patients between ages 3 months and 14 years, who presented to the pediatric emergency department and were diagnosed with severe community acquired pneumonia as per WHO guidelines, were included. All patients fulfilling inclusion criteria during the study period were enrolled consecutively.

There were three primary objectives of the study, which included (1) assessing the pattern of CXR abnormalities among children with a clinical diagnosis of severe community acquired pneumonia; (2) assessing the level of agreement among senior radiologists (MD + Radiology) on the WHO classification of CXR findings among children with severe community acquired pneumonia; and (3) assessing the determinants of duration of hospitalization of children with severe community acquired pneumonia.

### 2.2. Exclusion Criteria

Children with congestive heart failure, hospitalization within 14 days of a prior hospitalization episode or onset of pneumonia within 4 days of hospitalization, those diagnosed with tuberculosis, and children with foreign body aspiration or aspiration pneumonia were excluded from participating in the study.

### 2.3. Diagnosis and Management of Severe Community Acquired Pneumonia

In this study, the diagnosis of severe community acquired pneumonia was considered if the children presented with cough and/or with tachypnea defined by respiratory rate count of more than cut off for age (i.e., >/= 50 for those between ages of 3 months and 1 year; >/= 40 for children between the ages of 1-5 years) and the presence of one of intercostal and/or subcostal recessions, chest in-drawing, grunting, cyanosis, or altered mental status [5].

Treatment of severe community acquired pneumonia involves respiratory support—including oxygen administration via a nasal catheter or prong, antipyretics if fever >38.5°Celsius and antibiotics—intravenous crystalline penicillin in four divided doses until the child can take oral antibiotics. Additionally, any environmental, nutritional, or socioeconomic risk factors are carefully assessed and modifiable factors are addressed.

### 2.4. CXR Diagnosis of Pneumonia

Based on the WHO guidelines [8], the standard criteria for the radiologic diagnosis of pneumonia in a two view plain chest radiograph are defined as follows:

 
*Endpoint consolidation*: a dense or fluffy opacity that occupies the whole lobe of the lung or the entire lung often containing air bronchogram.
 
*Nonend point consolidation:* linear and patchy densities in a lacy pattern involving both lungs, featuring peribronchial thickening and multiple areas of atelectasis.
 
*Pleural effusion:* presence of fluid in the lateral pleural space evidenced by obliterating the costophrenic angle between the lung and chest wall.

Initially, CXR was independently read by two senior radiologists (MD + 3-Year Radiology Specialty)—referred to as* R1* and* R2*—following a checklist adopted from the WHO guidelines (Table 1). Findings and conclusion in which both radiologists agreed were taken as final and did not require reading by a third senior radiologist,* R3* who was used as a tie breaker for the study. Chest X-ray films with discordant readings in the initial evaluation were arbitrated by* R3*. The finding which was reported by two radiologists (*R1* and* R3* or* R2* and* R3*) was considered as a final CXR finding.

### 2.5. Data Collection Procedures

Every patient admitted to the emergency unit was registered in our study registration form by trained data collector nurses. At enrolment, through medical chart review and use of structured questionnaires, we collected dependent variables like demographics (age, sex, and address) of the child; clinical presentation (cough, fever, difficulty breathing, grunting, cyanosis, convulsions, inability to feed, and change in level of consciousness), previous history of similar problem, immunodeficiency (HIV, malnutrition, and diabetes mellitus), physical examination (vital signs, nutritional status, intercostal or subcostal retraction, nasal flaring, chest in-drawing, wheezing, crepitations, bronchial breath sounds, cyanosis, signs of rickets, and mental status), and laboratory results (white blood cell count, absolute neutrophil count, hemoglobin, platelet count, C-reactive protein, erythrocyte sedimentation rate, and blood culture). The children were followed during their stay in the hospital for evaluation of oxygen requirement, antibiotics therapy, feeding, persistence of fever, tachypnea, and duration of hospital stay and treatment outcome.

Nutritional assessment was done using the WHO recommended growth curves [25]. Z-scores were used to classify nutritional status.

Questionnaires were completed by trained data collection nurses. The study nurses accompanied patients to the radiology department for CXR, and films were read in sequence by* R2, *followed by* R1*.* R3* read all the films with disagreements at the end of data collection. Each radiologist was asked to complete the checklist of comments and findings. The readings by* R1* and* R2 *of the CXR films were used by the treating clinicians for patient care. We ensured that the study procedures did not delay patient treatment in any way.

### 2.6. Data Analysis

Descriptive statistics were analyzed and presented as frequency (percentage), mean (standard deviation), and median (interquartile range). The association of sociodemographic, clinical, and laboratory findings with CXR findings or CXR diagnosis of pneumonia was assessed using Fisher's exact test for categorical variables and Mann-Whitney U test. Nonparametric tests were used as we had a small sample size and the data were not normally distributed. Agreement between the two radiologists (*R1* and* R2*) was assessed using the Cohen's kappa coefficient.

Predictors of duration of hospital stay were assessed using a linear regression model. Multiple linear regression was used to assess the independent predictors which could significantly explain the variability in the duration of hospital stay among children with severe pneumonia. Variables which on bivariate analysis showed significance at a level of p<0.2 were included in the multiple linear regression model. P-values of <0.05 were considered as being statistically significant in the multiple linear regression model. SPSS for windows version 22 was used to analyze the data.

### 2.7. Ethical Consideration

Ethical approval was obtained from the Ethical Review Board of Hawassa University College of Medicine and Health Sciences and permission for data collection was obtained from HUCSH- Manager and Head of Department of Pediatrics. After brief explanation of the purpose of the study, informed consent was obtained from parents/guardians/or care givers of eligible children. Confidentiality was assured by excluding participant names and other identifiers from the analytic database. Participants had the right not to participate or withdraw from the study at any point. Patients judged unstable to undergo CXR were excluded from the study.

## 3. Results

### 3.1. Sociodemographic and Baseline Characteristics

A total of 122 children with severe pneumonia were followed from admission to discharge, loss to follow-up, or death. The median age of the cohort was 10.0 months IQR (6.75-24.0 months); 76 (62.3%) were male. The majority of the children, 77 (63.1%) were from the Southern Nations, Nationalities, Peoples' Region; 26 (21.3%) and 19(15.6%) were from the neighboring Oromia region and Hawassa city, respectively. Twenty-two (18%) children had been hospitalized previously. Antibiotic treatment at admission consisted of crystalline penicillin (56, 45.9%), ceftriaxone (64, 52.5%), and a combination of crystalline penicillin and ceftriaxone (2, 1.6%).

### 3.2. Radiologic Evidence of Clinically Diagnosed Severe Pneumonia

Over half (63, 51.6%) of the children diagnosed with severe pneumonia did not have radiologic evidence of pneumonia according to WHO CXR criteria. The only difference in clinical presentation between children with abnormal CXR and those with normal CXR was a higher body temperature at admission among those with radiologically confirmed pneumonia (p=0.033). Although not statistically significant, white cell count and hemoglobin levels were higher in children with normal CXR, and platelet counts were higher in those with abnormal CXR findings (Table 2).

### 3.3. Factors Associated with Duration of Hospitalization

The majority 118 (96.7%) were discharged home, whereas 3 (2.5%) absconded against medical advice and 1 (0.8%) died. The child that died was 8 months old who presented with fast breathing, cough, fever, and grunting of 3 days' duration. The median length of hospitalization was 3 days (IQR 2.75–4) days. On bivariate analysis, height-for-age z-score (p=0.027) and hemoglobin level (p=0.006) were associated with prolonged hospital stay (Table 3). However, on multiple linear regression modelling, hemoglobin was the only independent variable associated with prolonged hospitalization (p=0.007; Table 4).'

### 3.4. Agreement between Radiologists in Interpreting CXR Findings of Children with Severe Pneumonia

Radiologic diagnostic readings by* R1* and* R2* were not significantly different in interpretation of atelectasis, fibrosis, pleural thickening, and hyperinflation in children with severe pneumonia. Comment on film quality as adequate for reading was 74.6% by* R1* and 69.7% by* R2* (kappa=0.634 and p-value≤0.001). Presence of consolidation and haziness was reported in 22.1% and 9.8% (kappa=0.435, p-value≤0.001); 9.8% and 2.5% (kappa=0.0375, p-value=0.001), respectively, by* R1* and* R2* (Table 5). Overall, 43 (35.2%) CXR films required arbitration by* R3*.

## 4. Discussion

The present study evaluated the role of CXR for the diagnosis of severe pneumonia by radiologists in a cohort of children hospitalized at HUCSH, Hawassa, Southern Ethiopia. Evidence based definitive diagnosis assists clinicians to rationalize treatment options, so as to prevent prolonged hospitalization and overburdening of healthcare services and minimize cost to the public health sector.

In this study, the only clinical finding that discriminated significantly between children with radiographically confirmed pneumonia and those with normal CXR was degree of fever. Higher body temperature has been associated with delay in achieving clinical stability, although pattern of fever was not associated with radiologic findings [26].

The WHO has developed a clinical case definition of pneumonia for health care workers who work in settings with limited diagnostic capabilities [5]. Over the ensuing three decades, this definition has served as the primary means of identifying pneumonia among children in low and middle income countries. However, studies examining the specificity of this definition for the presence of radiographic pneumonia report discordant readings by radiologists. Only about 44% of more than 7,800 children meeting criteria for WHO-defined severe pneumonia in The Gambia had radiographic findings [27, 28]. Consistent with these, the current study found that radiologic evidence was found in 51.6% of children who were clinically diagnosed with WHO-defined severe pneumonia.

Discordant interpretation of CXR by radiologists may be due to variability in the use of the WHO CXR scoring system by health professionals or variation in the interpretation of chest radiographs by radiologists. Use of lung ultrasound has been shown to supplement CXR in the confirmation of clinical pneumonia and improves diagnostic yield of pneumonia in resource limited settings [29]. Results of our study suggest that there is a need for improved interpretation of CXRs by radiologists, and this would be anticipated to be even more necessary for clinicians that lack formal radiologic training.

Our findings on agreement between radiologists on most CXR findings were much lower than results reported by studies from western settings. For instance, a Spanish study found a concordance of 93.1% between radiologists for CXR interpretation (kappa=0.8) [30]. Another study from Brazil reported a concordance of 86.7% (kappa=0.683) with one radiologist reporting consolidation in 32.9% of CXRs whilst the other radiologist reported consolidation in 28.3% [31]. These findings are much better than the findings of the current study which found a statistically significant lack of concordance between radiologists for consolidation (kappa=0.435), infiltration (kappa=0.267), haziness (kappa=0.375), and effusion (kappa=0.658). The significant difference in interpretation of CXR between radiologists in our study may have been due to differences in training and experience between the specialists.

Proper diagnosis and treatment of pneumonia in children are associated with less hospitalization and lower cost to families and the healthcare setting. The median duration of hospitalization in our study was 3 days (IQR: 1–4 days) which is similar to a report from a high income setting in the Netherlands, where hospitalization ranged from 3 to 6 days [32]. Age, deranged vital signs, chest in-drawing, and infiltrates on radiography were the strongest predictors of severity of pneumonia in children [33, 34]. Malnutrition is associated with longer hospital stay due to the poor immune status, which could lead to other complications [35, 36]. Our findings showed that hemoglobin level was an independent predictor of duration of hospitalization. However, the current study did not reveal any relationship between nutritional status, physical findings, and duration of hospitalization.

The study has several strengths including the prospective design and the high burden setting. However, the results could have been more representative if more than one center were included, especially in an effort to address district and primary hospitals which might have different patient populations. Due to the low mortality rate, we did not have sufficient power to evaluate the association between chest radiographic evidence of pneumonia classification and mortality as an outcome in multivariable analysis.

In conclusion, this study revealed that the level of agreement between the trained radiologists in interpreting CXR findings of children with a clinical diagnosis of severe pneumonia was alarmingly low, particularly for commonly expected findings in cases of pneumonia. There is a low sensitivity of CXR for the diagnosis of childhood pneumonia. Interventions such as training, preparation of guidelines and other measures to build capacity for competence in interpretation of pediatric CXRs would be anticipated to improve radiologic diagnosis at our facility. Hemoglobin levels were found to be an independent predictor of prolonged hospitalization in our study, which suggests a possible role for hemoglobin analysis to predict outcome in our setting.

## Figures and Tables

**Table 1:** #**Form 2. CXR Assessment Checklist**. **Radiologist**: Please complete the following table for each CXR film.

SN	Parameter	Response	Comment
1	Patient ID	——————	

2	Radiologist Code	(1) R1 □ (2) R2 □ (3) R3 □	

3	Chest X ray code	——————	

4	Film Quality	(1) Adequate □ (2) Sup optimal □ (3) Not interpretable □	

5	Consolidation	(1) Yes □ (2) No □	If yes, location——————

6	Infiltration	(1) Yes □ (2) No □	If yes, location——————

7	Haziness	(1) Yes □ (2) No □	If yes, location——————

8	Pleural effusion	(1) Yes □ (2) No □	If yes, location——————

9	Atelectasis	(1) Yes □ (2) No □	If yes, location——————

10	Fibrosis	(1) Yes □ (2) No □	If yes, location——————

11	Pleural thickening	(1) Yes □ (2) No □	

12	Hyperinflation	(1) Yes □ (2) No □	

13	Index		

**Table 2:** Demographic and clinical characteristics of children with severe pneumonia in Hawassa University Comprehensive Specialized Hospital.

**Baseline data**	**Radiologic evidence of pneumonia**		**P-value**
	**Present (n=59)**	**Absent (n=63)**	
Age (months), Median (IQR)	10 (8-24)	10 (5-24)	0.550*∗*
Sex, Male (%)	40 (67.8)	36 (37.1)	0.264†
Duration of illness (days), Median (IQR)	3 (1-4)	3 (2-5)	0.269*∗*
Fever, Yes (%)	47 (46.1)	55 (53.9)	0.329†
Grunting, Yes (%)	26 (44.1)	32 (50.8)	0.474†
Vomiting, Yes (%)	22 (37.3)	24 (37.1)	-
Previous admission, Yes (%)	12 (20.3)	10 (15.9)	0.639†
Indoor cooking, Yes (%)	35 (59.3)	31 (49.2)	0.281†
Wheezing, Yes (%)	57(96.6)	54 (85.7)	0.055†
Crepitation, Yes (%)	50 (84.7)	56 (88.9)	0.595†
Mentation, Yes (%)	58 (98.3)	61 (96.8)	-
Respiratory rate, Median (IQR)	68 (60-76)	64 (54-70)	0.060*∗*
Pulse rate, Median (IQR)	148 (140-160)	146 (136-152)	0.076*∗*
Temperature (°C), Median (IQR)	38.1 (37.8-38.7)	37.8 (37.2-38.5)	**0.033** **∗**
SaPO2, Median (IQR)	85.0 (78.0-89,0)	85.5 (82-81.25)	0.187*∗*
White Blood Cell count, Median (IQR)	9,900 (7625-13,425)	10,000 (7,000-13,300)	0.998*∗*
Platelet count, Median (IQR)	352,000 (231,000-417,750)	317,000 (248,000-412,000)	0.724*∗*
Hemoglobin, Median (IQR)	11.2 (9.65-12.59)	11.8 (10.6-12.6)	0.224*∗*
Weight-for-Age Z-score, Median (IQR)	-0.85 (-1.73-0.50)	-0.15 (-1.11-0.71)	0.067*∗*
Height-for-Age Z-score, Median (IQR)	-0.61 (-2.01-0.76)	-0.25 (-1.29-1.06)	0.144*∗*
Weight-for-Height Z-score, Median (IQR)	-0.46 (-1.93-0.89)	-0.29 (-1.30-0.52)	0.495*∗*
BAZ, Median (IQR)	-0.5 (-1.93-0.80)	-0.43 (-1.55-0.63)	0.443*∗*

*∗*Mann-Whitney U test was used to compare the medians between groups; †Fisher's exact test was used for categorical variables; IQR, Interquartile Range; SaPO2, Saturation of Oxygen; BAZ, Body Mass Index-for-Age Z-score

**Table 3:** Bivariate linear regression model of predictors of duration of hospitalization among children with severe pneumonia, Hawassa, Ethiopia.

**Variable **	**Coefficient**	***R*** ^***2***^	**P-value**
Age in months	-0.123	0.015	0.177
Sex, Male	0.022	0.000	0.810
Duration of illness in days	0.070	0.005	0.443
Presence of fever	-0.084	0.007	0.355
Presence of vomiting	-0.116	0.013	0.204
Presence of grunting	0.070	0.005	0.440
Previous admission	0.060	0.004	0.515
Indoor cooking	-0.004	0.000	0.969
Breast feeding	0.071	0.005	0.435
Pulse rate (count per minute)	0.115	0.013	0.207
Respiratory rate (count per minute)	-0.048	0.002	0.599
Temperature in Celsius	0.117	0.014	0.198
Saturation of Oxygen (SaPO2)	0.031	0.001	0.772
Presence of cyanosis	-0.047	0.002	0.611
Finding of crepitation crepitation	-0.029	0.001	0.755
Finding of wheezing	-0.016	0.000	0.862
Level of consciousness	0.123	0.015	0.178
White blood cell count (cells/mm^3^)	-0.140	0.020	0.126
Platelet count (cells/mm^3^)	-0.141	0.020	0.122
Hemoglobin (gm/dL)	-0.249	0.062	**0.006**
Height-for-age Z score	0.203	0.041	**0.027**
Weight-for-height Z score	-0.049	0.002	0.605
Weight-for-age Z score	0.108	0.012	0.241
Body Mass Index-for-Age Z-score	-0.035	0.001	0.703
CXR positive (based on WHO criteria)	0.002	0.000	0.979

**Table 4:** Results of multiple linear regression analysis of predictors of duration of hospitalization among children with severe pneumonia, Hawassa, Ethiopia.

**Variable**	**Duration of hospitalization**≠		
	**Beta**	**T**	**p-value**
Age in months	0.051	0.544	0.588
Temperature in Celsius	0.098	1.059	0.292
Loss of consciousness	-0.052	-0.587	0.558
White blood cell counts per mL	-0.129	-1.420	0.159
Platelet counts per mL	-0.103	-1.128	0.262
Hemoglobin, g/dL	-0.252	-2.727	0.007
Height for ages z-score	0.176	1.862	0.065

≠Adjusted R^2^ = 0.081; p-value = 0.022.

**Table 5:** Agreement between senior radiologists in reading chest x-ray films of children with severe pneumonia, Hawassa, Ethiopia.

**Finding **	***R1*** **N (**%**)**	***R2*** **N (**%**)**	**Concordance**‡ **N (**%**)**	***R3*** **Total **n**/ **N, %	**Kappa** **∗**	**p-value**†
Film quality, %Adequate	91 (74.6)	85 (69.7)	103 (85.5)	28/43, 65.1	0.634	**< 0.001**
Consolidation present	27 (22.1)	12 (9.8)	101 (84.2)	13/41, 31.7	0.435	**< 0.001**
Infiltration present	20 (16.4)	13 (10.7)	99 (82.5)	6/41, 14.6	0.267	**0.008**
Haziness observed	12 (9.8)	3 (2.5)	111 (92.5)	2/41, 4.9	0.375	**0.001**
Effusion seen	3 (2.5)	3 (2.5)	118 (98.3)	0/41, 0	0.658	**0.001**
Atelectasis detected	0	0	120 (100)	-	-	-
Fibrosis present	0	0	120 (100)	-	-	-
Pleural thickening seen	0	0	120 (100)	-	-	-
Hyperinflation observed	1 (0.8)	0	120 (100)	-	-	-
WHO Diagnosis of abnormal CXR	58 (47.5)	30 (24.6)	86 (71.7)	21/41, 51.2	0.395	**≤ 0.001**

†Fisher's exact test; CXR, chest X-ray; *∗*agreement between *R1* and *R2*; ‡concordance between *R1* and *R2*.

## Data Availability

The data used to support the findings of this study are available from the corresponding author upon request.
